# Retinol Promotes *In Vitro* Growth of Proximal Colon Organoids through a Retinoic Acid-Independent Mechanism

**DOI:** 10.1371/journal.pone.0162049

**Published:** 2016-08-26

**Authors:** Taichi Matsumoto, Wakana Mochizuki, Yoichi Nibe, Shintaro Akiyama, Yuka Matsumoto, Kengo Nozaki, Masayoshi Fukuda, Ayumi Hayashi, Tomohiro Mizutani, Shigeru Oshima, Mamoru Watanabe, Tetsuya Nakamura

**Affiliations:** 1 Department of Gastroenterology and Hepatology, Graduate School, Tokyo Medical and Dental University, 1-5-45 Yushima, Bunkyo-ku, Tokyo 113–8519, Japan; 2 Department of Advanced Therapeutics for GI Diseases, Graduate School, Tokyo Medical and Dental University, 1-5-45 Yushima, Bunkyo-ku, Tokyo 113–8519, Japan; University Medical Center Utrecht, NETHERLANDS

## Abstract

Retinol (ROL), the alcohol form of vitamin A, is known to control cell fate decision of various types of stem cells in the form of its active metabolite, retinoic acid (RA). However, little is known about whether ROL has regulatory effects on colonic stem cells. We examined in this study the effect of ROL on the growth of murine normal colonic cells cultured as organoids. As genes involved in RA synthesis from ROL were differentially expressed along the length of the colon, we tested the effect of ROL on proximal and distal colon organoids separately. We found that organoid forming efficiency and the expression level of *Lgr5*, a marker gene for colonic stem cells were significantly enhanced by ROL in the proximal colon organoids, but not in the distal ones. Interestingly, neither retinaldehyde (RAL), an intermediate product of the ROL-RA pathway, nor RA exhibited growth promoting effects on the proximal colon organoids, suggesting that ROL-dependent growth enhancement in organoids involves an RA-independent mechanism. This was confirmed by the observation that an inhibitor for RA-mediated gene transcription did not abrogate the effect of ROL on organoids. This novel role of ROL in stem cell maintenance in the proximal colon provides insights into the mechanism of region-specific regulation for colonic stem cell maintenance.

## Introduction

The inner surface of the colon is lined with simple columnar epithelium structurally organized into crypts. The epithelium continues to self-renew throughout the lifetime, fueled by perpetual and rapid cellular turnover of *Lgr5*+ adult stem cells located near the base of those crypts [1, 2]. The fate determination of those colonic stem cells is, as is the case with small intestinal stem cells, governed by multiple regulatory signals such as the Wnt, bone morphogenic protein (BMP), Notch signaling pathway, and receptor tyrosine kinases [2, 3].

The proliferative activity of colonic stem cells can be reconstituted *in vitro* by using the organoid culture system that has recently emerged as a powerful tool for studying stem cell biology. Sato et al. first described the system, which allows long-term expansion of murine small intestinal cells as a structure consisting of crypt domains harboring *Lgr5*+ stem cells and also a domain lined by differentiated cell types [4]. Importantly, this condition requires Rspo1 (Wnt agonist), Noggin (BMP inhibitor) and Epidermal Growth Factor (EGF), and therefore faithfully recapitulates the functional importance of those signaling pathways for *in vivo* maintenance of small intestinal stem cells. The organoid culture system was shown to be applicable to colonic epithelial cells when the culture medium is supplemented with Wnt ligands in addition to the factors required for small intestinal cell culture [5].

Using a slightly different combination of growth factors and extracellular matrices, we have also developed a method to culture colonic stem cells [6]. Murine colonic cells were shown to grow almost in perpetuity as spherical organoids in the presence of Wnt3a, Rspo1, Noggin, EGF, Hepatocyte Growth Factor (HGF) and Bovine Serum Albumin (BSA) even under serum-free conditions [6]. The cells expanded by this method were shown to be capable of regenerating normal colonic epithelial tissues when transplanted into mice in which colonic mucosal injuries were induced [6]. This indicates that the cells in organoid cultures preserve many aspects of their original features *in vitro*, building a rationale for the use of this culture system to assess the response of colonic stem cells to various stimuli and identifying underlying mechanisms that drive those responses.

Retinol (ROL) is the alcohol form of vitamin A, which controls proliferation and differentiation in various types of cells [7]. ROL is ubiquitously present in the circulating blood and delivered to different cell types with different specificities [8]. In target cells, ROL is metabolized to retinoic acid (RA) in two steps of oxidation. ROL is first oxidized to retinaldehyde (RAL) by either alcohol dehydrogenases (ADH1, ADH5, and ADH7) [9–11] or retinol dehydrogenases (RDH1 and RDH10) [12, 13]. RAL is then further oxidized to RA by members of the aldehyde dehydrogenase family (ALDH1A1, ALDH1A2, and ALDH1A3) [14–17]. Most of the actions of ROL are generally thought to be mediated primarily by RA, which regulates gene transcription by functioning as a ligand for the retinoic acid receptors (multiple isoforms of RARα, β, and γ) and the retinoid X receptors (multiple isoforms of RXRα, β, and γ) [7, 9, 18]. This ROL-RA pathway is known to control cell fate decisions and the maintenance of various types of stem cells. Although the pathway acts to induce differentiation in many stem cell types [7], it also promotes proliferation in some types of stem cells such as embryonic stem cells [19] and germline stem cells [20].

With regard to colonic epithelial cells, a few studies described the effect of ROL on the growth of human colorectal cancer-derived cell lines [21–23]; however, little is known about whether ROL plays a role in the maintenance of normal colonic epithelia and their stem cell populations. Therefore, we sought to address this question by using the system that we developed [6] to culture primary non-transformed colonic cells *in vitro*.

## Materials and Methods

### Mice

C57BL6 male mice at 8 to 12 weeks of age were used in this study. Mice were bred under standard conditions at our university. We used tissue samples that were removed from mice immediately after they were sacrificed by cervical dislocation. All animal experiments were performed with the approval of the Institutional Animal Care and Use Committee of TMDU.

### Colon Organoid Culture

Colonic crypt isolation was performed according to the method described previously [6]. When indicated, crypts were separately isolated from proximal and distal halves of the colon. Briefly, after extensive washing, colonic tissues minced into small pieces were incubated in Dulbecco’s modified eagle medium (DMEM) containing 1% FBS, 500 U/ml collagenase XI (Sigma), 0.4 U/ml dispase (Roche), and 1 mM dithiothreitol (DTT) at 37°C for 20 min. The crypts were further purified by mechanical disruption and density gradient centrifugation. A total of 400 crypts were suspended in 40 μl of the collagen type I solution (Nitta Gelatin Inc.) and placed in 24-well plates. After polymerization, 500 μl of Advanced DMEM/F12 containing 1% BSA (Sigma A9576), 30 ng/ml mWnt3a (R&D Systems), 500 ng/ml mRspo1 (R&D Systems), 20 ng/ml mEGF (Peprotech), 50 ng/ml mHGF (R&D Systems), and 50 ng/ml mNoggin (R&D Systems) were added to each well. The medium was changed every 2 days. The organoids were cultured for 6 days and then used for the following analysis with single-cell passage procedures.

### Single Cell Passage

For single-cell passage, the collagen gel was digested in DMEM containing collagenase type XI (Sigma) at 37°C for 5 min. The released organoids were washed in PBS containing 0.5% BSA. The organoids were digested in 4 ml of TrypLE Express (Thermo Fisher Scientific) at 37°C for 5 min, and then vigorously shaken to obtain disaggregated cells. Single cell preparation was verified by microscopic inspection and viable cells were counted in a hemocytometer by method of Trypan blue exclusion. Cells were then seeded so that 1 x 10^4^ cells were present in 40 μl of the collagen type I gel in each well at the start of culture. When necessary, retinol (Sigma R7632), *all*-*trans*-retinal (retinaldehyde) (Sigma R2500) and *all*-*trans*-retinoic acid (Sigma R2625) reconstituted in ethanol were added to the culture at indicated doses after passage. AGN 193109 (Santa Cruz Biotechnology, sc210768), reconstituted in DMSO, was added to the medium at 1 μM together with ROL or RA as indicated. The organoids cultured in this way were recovered from the collagen gel on Day 5 of culture and used for another round of culture after single cell passage.

### Determination of Organoid Forming Efficiency

Organoid forming efficiency was evaluated by initiating culture with 1x 10^4^ cells per well after single cell passage. On Day 5 of culture, Z-stacks of phase-contrast images were acquired at Z-steps of 30 μm on a microscope BZ-X710 (KEYENCE). In most cases, depth of observation up to 1 mm was sufficient to cover the entirety of the collagen type I gel in each well. We counted the number of organoids that were clearly visible as round cystic structures on Z-projection images created by the BZ-X710 system. Data were collected for triplicate wells for each of 3 independent experiments (n = 9). The diameter of organoids was analyzed on Z-projection images created as described above. Squares 1.5 mm per side were placed at the center of the images of 9 individual wells (triplicate wells from one of three donor samples). The diameter of each organoid included in the squared area was measured and used for quantitative analysis. Statistical significance was determined by Student’s t test (p < 0.05).

### Semi-quantitative RT-PCR

Semi-quantitative RT-PCR was performed in a standard fashion. Aliquots of 300 ng of total RNA were used for cDNA synthesis in 21 μl of reaction volume. One microliter of cDNA was used for the following RT-PCR. Primer sequences and the detail of reactions are listed in S1 Table. PCR products were separated on agarose gels and visualized using ImageLab (Bio-Rad).

### In Situ Hybridization (ISH)

ISH for *Aldh1a1* and *Aldh1a3* was performed as described previously [24]. The pcDNA3 plasmid containing a cDNA fragment of mouse *Aldh1a1* (nucleotides 65–1892; GenBank NM_013467.3) or *Aldh1a3* (nucleotides 2312–3225; GenBank NM053080.3) was constructed. Single-stranded, digoxigenin-labeled RNA probes were generated by an *in vitro* transcription system (Roche). Frozen sections of colonic Swiss rolls were rehydrated, treated with HCl, digested in proteinase K solution, postfixed, treated in acetic anhydride solution, and hybridized overnight at 65°C with probes. After extensive rinsing and washing, sections were then subjected to the immunohistochemistry by using alkaline phosphatase–conjugated anti-digoxigenin antibody (Roche). Sections were reacted with nitroblue tetrazolium/5-bromo-4-chloro-3-indolyl phosphate solution for color development. Images were acquired on a microscope BZ-X710 (KEYENCE). Images were processed using Adobe Photoshop software.

### Immunohistochemistry

Colonic tissues were fixed overnight at 4°C in 4% paraformaldehyde, sequentially dehydrated in 10, 15 and 20% sucrose in PBS, and embedded in OCT compound (Tissue Tek). Colonic organoids were fixed together with surrounding collagen type I gel and processed in the same manner. Frozen sections of 8-μm thickness were subjected to immunohistochemistry by using antibodies specific for ALDH1a1 (Abcam ab52492), ALDH1a3 (Abjent AP7847a), or Ki-67 (Dako Cytomation). In all immunofluorescence experiments, nuclei were counterstained with DAPI. Fluorescent images of sections were acquired using a microscope BZ-X710 (KEYENCE). If necessary, image processing was carried out using Adobe Photoshop software. For quantification of ALDH1a3+ and Ki-67+ cells, triplicate wells from one of three donor samples were used. On Day 5 after passage, sections of thirty different organoids were chosen from a series of sections obtained from each well. ALDH1a3+ or Ki-67+ cells were counted, and values are presented as a mean percentage to whole cell populations for sections of those 270 organoids. Statistical significance was determined by Student’s t test (p < 0.05).

## Results

**Fig 1:**
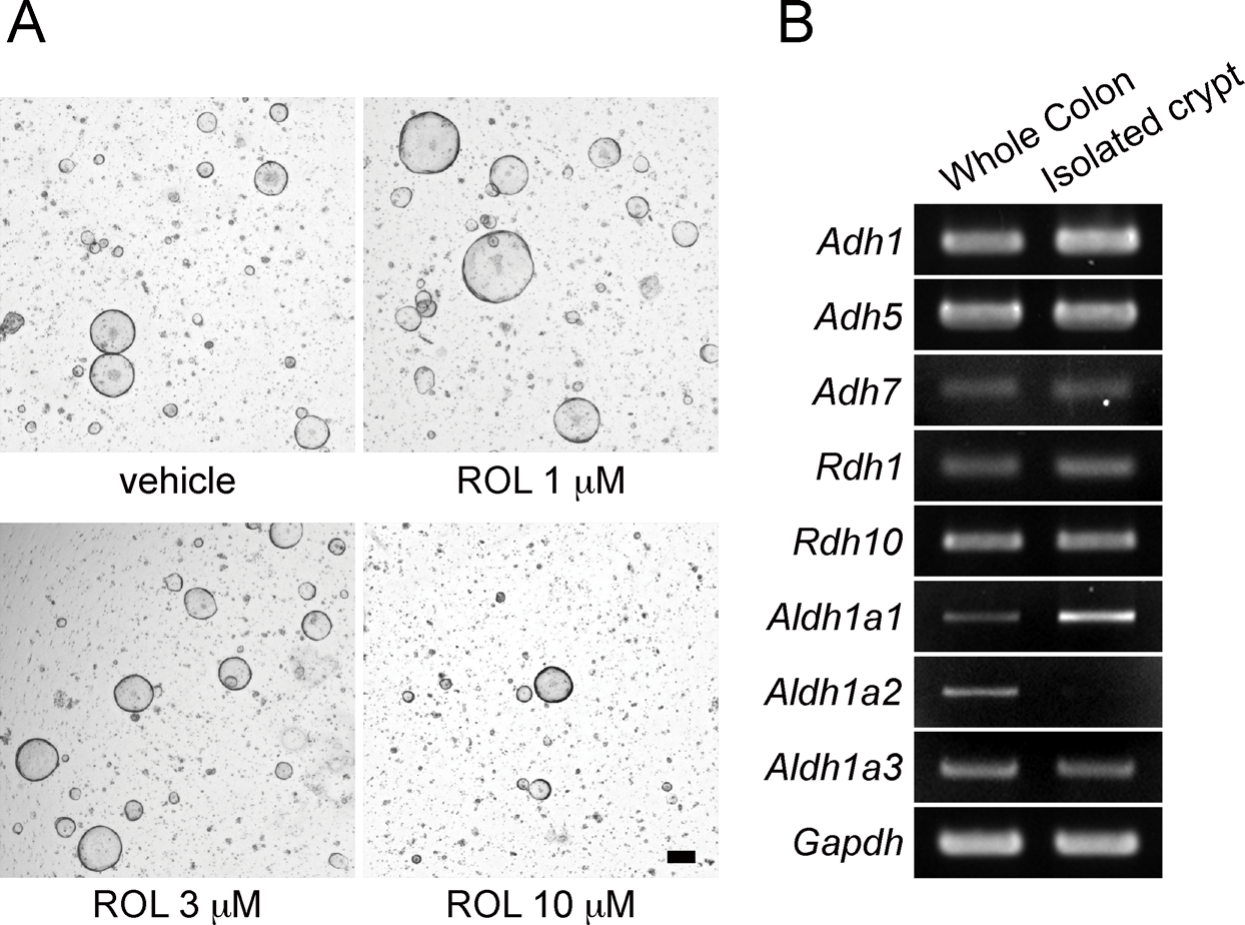
Expression of genes involved in ROL-RA pathway in colonic epithelia. (A) Crypts isolated from the entire length of the colon were cultured for 6 days. Organoids were then dissociated into single cells and further cultured in the presence of vehicle alone, or indicated concentrations of ROL. Phase-contrast images were acquired on Day 5 of culture. Representative images of three independent experiments are shown. Scale bar, 100 μm. (B) Total RNA was extracted from the whole colon or its epithelial compartment isolated as crypts. Semi-quantitative PCR was performed for the indicated genes. Representative data are shown for three independent experiments.

We first investigated whether retinol (ROL) has effects on the *in vitro* growth of epithelial cells that were isolated from the entire length of the mouse colon. We previously reported that, when colonic crypts were isolated and cultured as organoids, *Lgr5*+ stem cells increased in number over the first several days [6]. To test the effect of ROL on the growth and organoid forming efficiency of the stem cell population, colonic cells were cultured for 6 days after isolation to obtain sufficient number of stem cells. Next, the organoids were dissociated into single cells and the first-passage culture was initiated from this single cell population in the presence or absence of ROL. We found that, when various concentrations of ROL were added to the culture, cells treated with 1 μM or 3 μM of ROL formed a somewhat higher number of organoids as compared to those cultured in the absence of ROL (Fig 1A). In addition, some organoids treated with ROL (1 μM or 3 μM) appeared to have larger diameters than untreated organoids (Fig 1A). Cells treated with ROL at 10 μM (Fig 1A) or greater concentrations (not shown) showed a lower number of organoids than untreated controls. As the levels of ROL in human serum were reported to range from 0.5 to 2 μM [25], we presumed that the treatment with physiological concentrations of ROL would have a positive effect on the organoid forming efficiency of cultured colonic epithelial stem cells.

ROL is widely known to induce various biological effects in the form of retinoic acid (RA). To assess whether cultured colonic organoids contain the cellular machinery for converting ROL to RA, we investigated the expression of genes involved in the ROL metabolism. Semi-quantitative RT-PCR revealed that mRNAs encoding enzymes capable of catalyzing the first step of the reaction (*Adh1*, *Adh5*, *Adh7*, *Rdh1*, and *Rdh10*) were all present in the whole colon tissues as well as in the isolated crypts (Fig 1B). In regard to three ALDH1a family isoenzymes known to catalyze the second step of reaction, mRNAs for *Aldh1a1* and *Aldh1a3* were found to be present in both samples (Fig 1B). *Aldh1a2* mRNA was expressed in the whole tissue, but it was not detected in the epithelial compartment (Fig 1B). These observations indicated that the enzymes that regulate RA synthesis are readily expressed in the colonic epithelium, and this ROL-RA pathway might be related to the seemingly positive but moderate effects of ROL on the organoid forming efficiency of cultured colonic stem cells.

**Fig 2:**
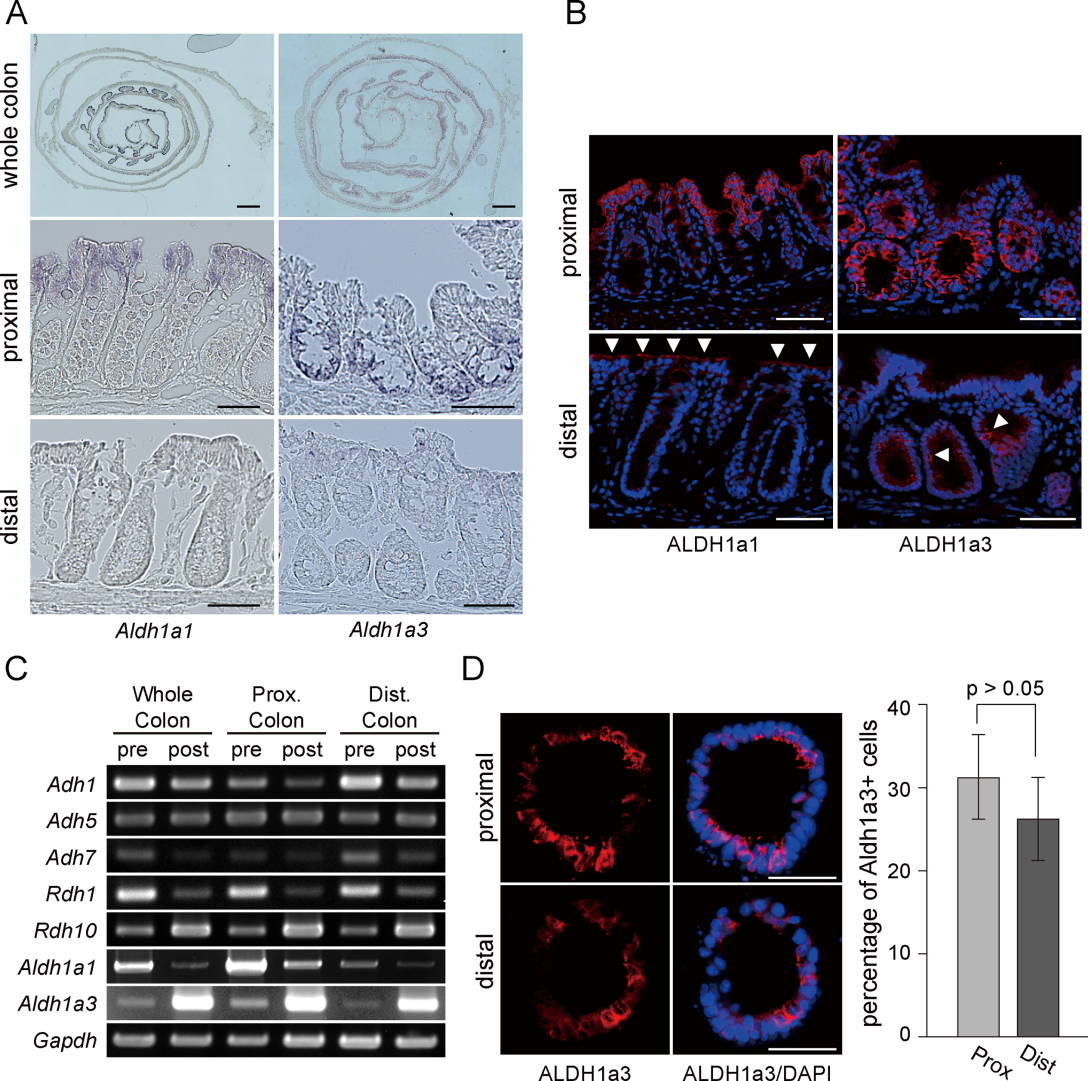
Enzymes involved in ROL-RA pathway are differentially expressed along the length of the colon. (A) Colonic Swiss rolls were assessed for expression of *Aldh1a1* (top left) and *Aldh1a3* (top right) genes by in situ hybridization. The proximal and distal portions of the colon are located inside and outside, respectively. Magnified views are shown for the proximal (middle) and distal colon (bottom). Representative images of three independent experiments are shown. Scale bars, 1 mm for images on the top and 50 μm for images on the middle and at the bottom. (B) Sections of the proximal (top) and distal (bottom) colon were assayed for protein expression of ALDH1a1 and ALDH1a3. Signals yielded by immunohistochemical staining (red) are shown in merged images with DAPI staining (blue). Representative data are shown for three independent experiments. Arrowheads show positive cells for ALDH1a1 or ALDH1a3 in the distal colon. Scale bars, 50 μm. (C) Colonic crypts were isolated from the entire length (whole colon), or from proximal (prox. colon) or distal (dist. colon) half of the colon. Crypts were then cultured for 6 days. Total RNA was extracted from the isolated crypts before (pre) or after the culture (post). Semi-quantitative PCR was performed for the genes indicated. Representative data are shown for three independent experiments. (D) Colonic crypts were separately isolated from proximal and distal colons and cultured as organoids. Frozen sections of organoids were subjected to immunohistochemistry for ALDH1a3 (left). Merged images with DAPI staining were also shown (right). Scale bars, 50 μm. Sections of 30 organoids were chosen from a series of sections obtained from each well (triplicate wells for each of three independent experiments) and the percentage of Ki-67+ cells to whole cell populations was quantitated (graph on the right). Values are presented as mean ± s.e.m. for those 270 sections.

Through the further analysis of genes involved in RA synthesis, we noticed intriguing expression patterns of *Aldh1a1* and *Aldh1a3* in the colon. Consistent with the RT-PCR data (Fig 1B), *in situ hybridization* (ISH) revealed that both genes were strongly expressed in the epithelial layer of the colon (Fig 2A). Interestingly, *Aldh1a1* and *Aldh1a3* showed distinct regional differences in distribution along the length of the colon; they were both expressed predominantly in the proximal colon and their expression levels declined in the distal colon and rectum (Fig 2A, top). In addition, these two genes were differently expressed along the crypt axis; *Aldh1a1* was preferentially expressed in the surface epithelium, while expression of *Aldh1a3* was relatively confined to the crypt bottom (Fig 2A, middle). The mucosa near the distal end of the colon exhibited low or undetectable expression of these two genes (Fig 2A, bottom). The characteristic distribution patterns of ALDH1a1 and ALDH1a3 were also observed at the protein expression level. Immunohistochemistry revealed that ALDH1a1 and ALDH1a3 were predominantly expressed in the proximal colon, while they appeared only in a few cells at low level in the distal part of the colon (Fig 2B). Again, ALDH1a1 was mainly located at the surface epithelium, whereas ALDH1a3 was detected at the lower part of the crypt (Fig 2B).

To confirm the unique distribution patterns of *Aldh1a1* and *Aldh1a3* in the colon, we performed semi-quantitative RT-PCR again with the epithelia separately isolated from the proximal and distal halves of the colon. In addition, in order to assess the gene expression changes during the culture, crypts of the whole colon and those from two colonic regions were independently cultured and their mRNAs were subjected to RT-PCR. In line with the ISH and immunohistochemistry data, the expression levels of *Aldh1a1* and *Aldh1a3* in pre-culture samples (isolated crypts) were confirmed to be significantly higher in the proximal colon crypts than those in the distal ones (Fig 2C). By contrast, *Adh1* and *Adh7* genes showed higher expression in pre-culture samples isolated from the distal colon compared with those from proximal one. Other genes involved in ROL metabolism (*Adh5*, *Rdh1* and *Rdh10*) showed no obvious regional differences between pre-culture crypts (Fig 2C). We also found in this experiment that, during the culture process, expression levels of *Adh1*, *Rdh1* and *Aldh1a1* were decreased, while those of *Rdh10* and *Aldh1a3* were increased in both proximal and distal colonic epithelia (Fig 2C). To investigate whether such mRNA expression changes during culture lead to alterations of protein expression, crypts from proximal and distal halves of the colon were separately cultured and processed for immunohistochemistry for ALDH1a3, which showed a significant increase in mRNA expression during culture (Fig 2C). It was shown that organoids derived from proximal and distal parts clearly contained ALDH1a3+ cells, which represented ~30% of total cells in both samples (Fig 2D). Considering that ALDH1a3 protein expression in the distal colonic tissue was observed in fewer cells than in the proximal one (Fig 2B), this suggested that ALDH1a3 expression increased in the course of culture not only at mRNA level but also at protein level, at least in distal colon organoids. We previously demonstrated that the culture protocol that we developed preferentially expands *Lgr5*+ stem cells [6]. Since *Aldh1a3* was expressed in the proliferative zone at the crypt bottom (Fig 2B & 2C), we assumed that the expression changes of *Aldh1a3*, and also other genes (*Aldh1a1*, *Adh1*, *Rdh1* and *Rdh10*), might be associated with alterations in cell-type composition during the organoid culture.

**Fig 3:**
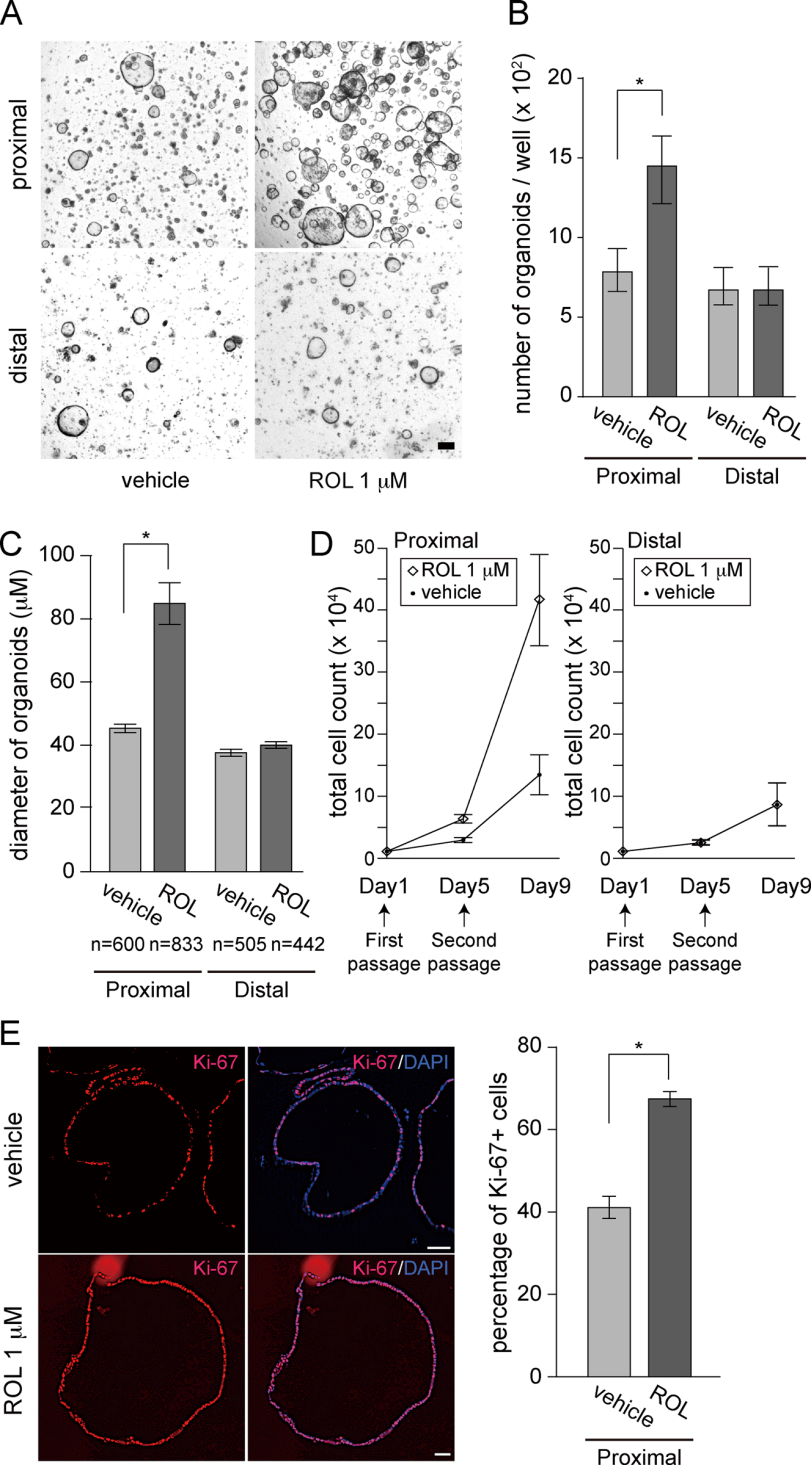
ROL enhances growth of proximal colon organoids but not of distal ones. (A) Proximal and distal colon organoids were separately subjected to single-cell passage. Respective cells were cultured in the absence (vehicle) or presence of ROL (1 μM) and phase-contrast images were acquired on Day 5. Representative images of three independent experiments are shown. Scale bar, 100 μm. (B) Single-cell passage and the following culture was initiated as in (A) at a density of 1 x 10^4^ cells/well. On day 5, round cystic organoids that formed in each well were counted as described in Materials and Methods. Values are presented as mean ± s.e.m. (n = 9). *, *P* < 0.05. (C) Proximal and distal colon cells were cultured as in (B). Diameters of organoids were measured on Day 5 of culture as described in Materials and Methods. Total number (n) of organoids analyzed in each group was shown at the bottom. Data are presented as mean ± s.e.m. (n = 9). *, *P* < 0.05. (D) Proximal and distal colon cells were cultured as in (B) at a density of 1 x 10^4^ cells/well (Day1). Triplicate wells from one of three independent donor samples were separately cultured. On Day 5, organoids were recovered and then the second passage was performed. Cell numbers were counted on Day 5 (at the time point of second passage) and Day 9. Data are presented as mean ± s.e.m. (n = 9). *, *P* < 0.05. (E) Proximal colon cells were cultured as in (B). On Day 5, frozen sections of organoids were subjected to immunohistochemistry for Ki-67 (left). Merged images with DAPI staining were also shown (right). Scale bars, 50 μm. The percentage of Ki-67+ cells to whole cell populations was quantitated (graph on the right) as described for Fig 2D. Values are presented as mean ± s.e.m. for those 270 sections. *, *P* < 0.05.

Given the observation that many genes involved in the ROL metabolism, most exemplified by *Aldh1a1* and *Aldh1a3*, exhibited regional differences in their expression along the colon, we thought that ROL might induce different responses between proximal and distal colon organoids. To test this idea, crypts were isolated separately from the two portions of the colon, cultured for 6 days, and passaged as single cells to test the effect of ROL (1 μM). We found that the number of ROL-treated proximal colon organoids was apparently greater than that of untreated organoids (Fig 3A). By contrast, organoids from the distal colon showed no difference between ROL-treated and untreated samples (Fig 3A). To quantitatively assess this finding, we counted the number of organoids that formed clearly discernible round cystic structures on Day 5 after passage. The organoid forming efficiency was significantly higher in ROL-treated proximal colon organoids compared with untreated controls (Fig 3B). Cells from the distal colon showed no difference in this assay between ROL-treated and untreated groups (Fig 3B). We also measured the size of organoids on Day 5. ROL-treated proximal colon organoids had a significantly larger average diameter than did those untreated with ROL (Fig 3C). Such a difference was not observed in distal colon organoids (Fig 3C). To directly assess the expansion of cell populations, we conducted the single-cell passage, initiated organoid culture and then recovered the whole cell population on Day 5 to count them. Under this condition of single-cell passage, the total cell count increased steadily in organoids even in the absence of ROL (Fig 3D). Of note, supplementation of culture medium with ROL (1 μM) significantly enhanced the increase of cell numbers in proximal colon organoids as compared to controls, and this trend continued for another 4 days when the single-cell culture procedure was repeated (Fig 3D, Day 9). To investigate whether the ROL-dependent increase in cell number was due to the enhanced cellular proliferative activity, we fixed the organoids and immunostained the sections for Ki-67 on Day 5 after passage. In proximal colon organoids cultured in the absence of ROL, Ki-67+ cells were scattered sparsely throughout the structure (Fig 3E, top left). By contrast, ROL-treated proximal colon organoids were lined by far more Ki-67+ cells (Fig 3E, bottom left). This was also confirmed by direct counting of Ki-67+ cells in those sections (Fig 3E, right).

**Fig 4:**
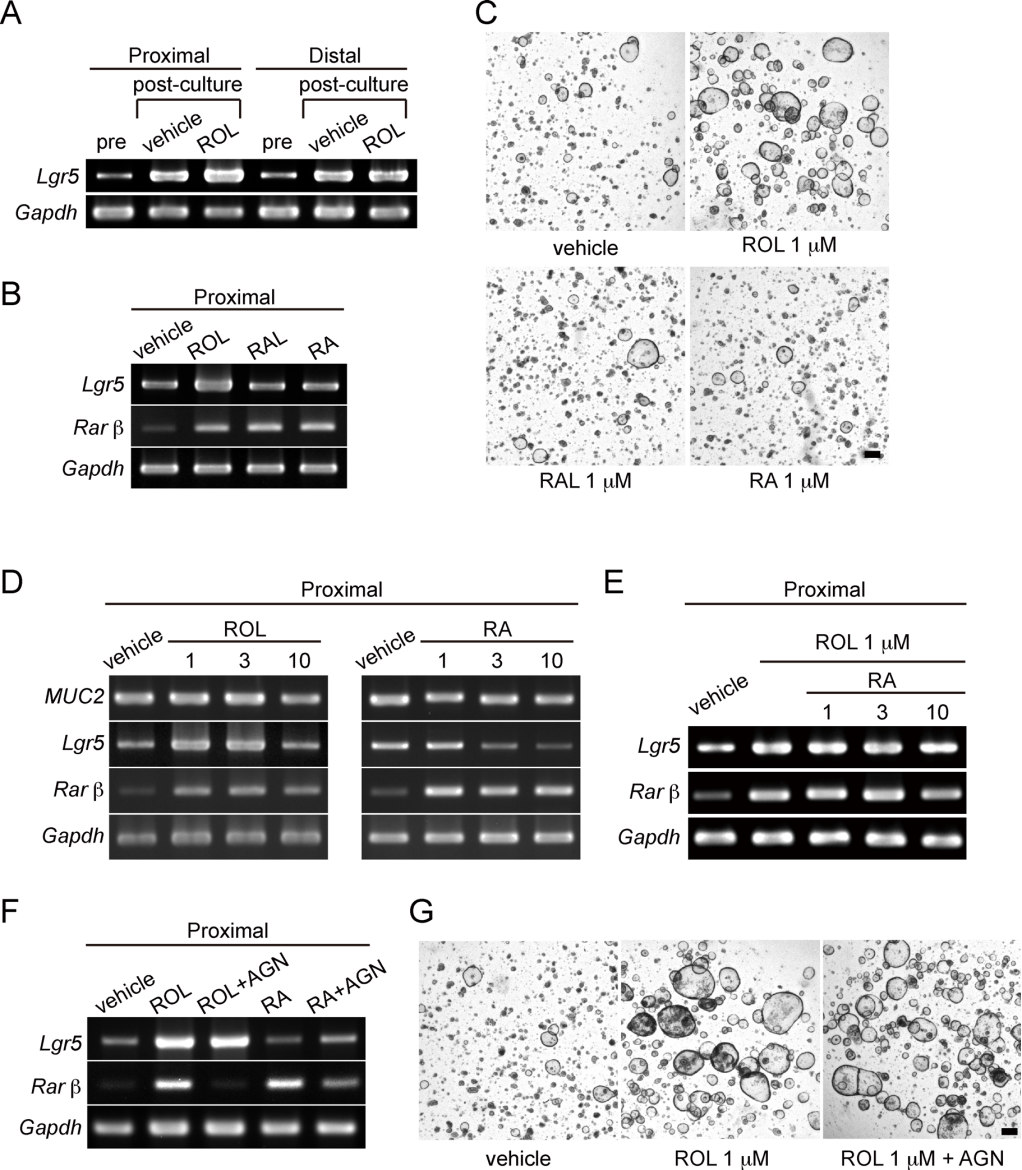
ROL-dependent growth promotion of proximal colon organoids involves an RA-independent mechanism. (A) Proximal and distal colon organoids were separately subjected to single-cell passage, and the following culture was initiated in the absence (vehicle) or presence of ROL (1 μM). Total RNA was extracted from the isolated cells before (pre) or after the culture (post). Semi-quantitative PCR was performed for *Lgr5*, a marker gene of colonic stem cells, and *Gapdh*. (B) After single-cell passage, proximal colon organoids were cultured either with ROL (1 μM), RAL (1 μM) or RA (1 μM) or left untreated (vehicle). Total RNA was extracted after the culture, and semi-quantitative PCR was performed for *Lgr5*, *Rarβ*, and *Gapdh*. (C) Proximal colon cells were cultured as in (B). Representative phase-contrast images on Day 5 are shown. Scale bar, 100 μm. (D) Proximal colon cells were cultured as in (B) either with different concentrations (1, 3, or 10 μM) of ROL or RA, or left untreated (vehicle). Total RNA was extracted on Day 5 of culture and semi-quantitative PCR was performed for *MUC2*, *Lgr5*, *Rarβ* and *Gapdh*. (E) Proximal colon cells were cultured in the absence (vehicle) or presence of 1 μM ROL. ROL-treated cells were co-treated with different concentrations of RA (1, 3, or 10 μM) or left untreated. Semi-quantitative PCR was performed as described in (B) for *Lgr5*, *Rarβ* and *Gapdh*. (F) Proximal colon cells cultured as in (B) were left untreated (vehicle) or treated with either ROL, ROL+AGN193109, RA, RA+AGN193109 all at a concentration of 1 μM. On Day 5 of culture, total RNA was extracted after the culture, and semi-quantitative PCR was performed for *Lgr5*, *Rarβ* and *Gapdh*. (G) Proximal cells were cultured as in (F) and phase-contrast images on Day 5 were acquired. Scale bar, 100 μm. Experiments for A-G were performed more than twice independently and representative images are shown.

We next investigated whether the expression of *Lgr5*, a marker gene for colonic stem cells, was influenced by ROL treatment. Proximal and distal colon cells were cultured for 5 days after single cell passage, and mRNA extracted from those cells before and after the culture was assessed by RT-PCR. It was shown that, even in the absence of ROL, expression of *Lgr5* was increased during culture in both the proximal and distal colon (Fig 4A), as a result of efficient expansion of the stem cell pool by this culture method [6]. Notably, treatment with ROL further augmented the induction of *Lgr5* gene expression in the proximal colon organoids, but this phenomenon was not observed in the distal organoids (Fig 4A). These data clearly indicate that the ROL-mediated acceleration of the growth rate of proximal colon organoids involves the stimulation of *Lgr5*+ stem cells.

To examine whether the ROL-RA pathway mediates the effect of ROL on organoid growth, proximal colon cells were treated either with ROL, RAL or RA (all at a concentration of 1 μM) after single cell passage. Organoids were collected on Day 5 of culture and their mRNA was analyzed. Unexpectedly, in contrast to the significant enhancement of *Lgr5* expression in ROL-treated cells, no obvious change was detected in RAL- or RA-treated organoids (Fig 4B). Meanwhile, expression of *Rarβ*, one of the target genes of RAR-mediated transcription, was clearly induced in organoids treated with any of the three forms of retinoids (Fig 4B). This indicated that RA synthesis from ROL or RAL, and also the RAR-dependent transcription are operational in cultured organoids. Microscopic observation also revealed that ROL promoted organoid growth of proximal colon organoids, while RAL and RA did not show such an effect (Fig 4C). These observations suggested that the ROL-dependent induction of *Lgr5* expression and growth enhancement in proximal colon organoids involved a mechanism distinct from the ROL-RA pathway.

Several studies have shown that ROL and RA suppress proliferation of human colon cancer cell lines [21–23, 26]. In addition, other reports demonstrated that RA induces expression of *MUC2*, a differentiation marker gene, in SW480 colon cancer cells [27, 28]. To investigate the effects of retinoids on differentiation of cultured colonic cells, we assessed the expression level of *MUC2* gene in organoids treated with various doses of ROL or RA, or left untreated (Fig 4D). Proximal colon organoids did not show changes in *MUC2* mRNA expression in response to any concentration (1, 3, or 10 μM) of ROL and RA, indicating that retinoid treatment does not drive differentiation of colonic cells in cultured organoids under this condition (Fig 4D). We also found with this experiment that proximal colon cells treated with 10 μM ROL, or with 3 or 10 μM of RA did not show enhanced growth as judged by microscopic analysis (data not shown). Consistently, *Lgr5* expression was up-regulated with 1 μM and 3 μM of ROL at a comparable level, whereas 10 μM ROL did not show such an effect (Fig 4D). In addition, the *Lgr5* level remained unchanged when the proximal organoids were treated with 1 μM RA, but was down-regulated by higher concentrations (3 or 10 μM) of RA. The RAR-mediated transcription seemed unlikely to be involved in this phenomenon, as the *Rarβ* induction seen with stimulation by 1 μM of these retinoids did not increase any further by higher doses (3 or 10 μM) of ROL or RA (Fig 4D). Although the mechanism of these suppressive action of high doses of ROL (10 μM) and RA (3 and 10 μM) on Lgr5 expression remains unclear; however, these results reinforced the notion that the growth-promoting effects of low dose ROL is independent from the direct action of RA.

In order to assess hierarchy relationship between opposing actions of low dose ROL and RA, we tested whether co-addition of RA (at 1, 3 or 10 μM) might affect the induction of *Lgr5* expression by 1 μM ROL. Since further induction of *Rarβ* was not observed in the co-presence of ROL and RA (Fig 4E), the transcription activity of RARs appeared to reach the maximum level by 1 μM or lower concentration of ROL or RA, which was also supported by the data shown in Fig 4D. Of note, addition of RA did not counteract the ROL-dependent up-regulation of *Lgr5* even when used at 3 or 10 μM (Fig 4E). These data indicated that, when ROL and its metabolite RA coexist, ROL functions as an independent and dominant factor to regulate the expression of *Lgr5*.

To further confirm this independent action of ROL, we treated the proximal colon organoids cultured in the presence of ROL (1 μM) or RA (1 μM) with a pan-RAR antagonist, AGN 193109 [29]. The action of AGN 193109 was verified as it completely abrogated the ROL- and RA-dependent *Rarβ* gene induction (Fig 4F). By contrast, AGN 193109 did not cancel the ROL-dependent enhancement of *Lgr5* expression (Fig 4F). Furthermore, the organoid forming efficiency raised by ROL in proximal colon organoids was not affected by co-incubation of the culture with AGN 193109 (Fig 4G). These collective data indicate that ROL-dependent stimulation of *Lgr5*+ stem cells of the proximal colonic epithelium is mediated by an RA-independent mechanism.

## Discussion

We showed in this study that several enzymes that are involved in RA synthesis are differentially expressed in epithelia along the length of the mouse colon *in vivo*. In particular, among these enzymes, we confirmed that *Aldh1a1* and *Aldh1a3* are predominantly expressed in the proximal colon epithelium by ISH and immunohistochemial analyses. A previous study investigated expression patterns of *Aldh1a1*, *Aldh1a2* and *Aldh1a3* in the mouse intestine [30]; however, it only described their expression patterns in the fetal small intestine. Therefore, the present study is the first to show the distribution patterns of ALDH1 genes and proteins in the colonic epithelium of adult mice. Importantly, detection of uneven expression patterns of ALDH1a1 and ALDH1a3 in the colon epithelium triggered our study to assess the effect of ROL on proximal and distal colon organoids separately, which led us to demonstrate that ROL has potent activity to promote the growth of proximal colon organoids, but not of distal ones. This finding suggests that the stem cells located in distinct regions of the colon involve distinct mechanisms for their proliferation.

It is generally accepted that ROL exerts its biological effect on differentiation and proliferation of target cells through the action of its active metabolite, RA [9]. Contrary to this, we showed that ROL enhances the growth of proximal colon organoids through an RA-independent mechanism. Treatment of colonic organoids with RAL or RA did not result in enhanced growth and *Lgr5* gene induction. In addition, co-incubation of ROL-treated cells with an RAR antagonist did not suppress the enhanced *Lgr5* expression or organoid forming efficiency. Previous studies have shown that ROL supports the self-renewal of mouse embryonic stem cells (ESCs) in long-term cultures even in the absence of feeder cells [31]. The same authors further reported that this action of ROL is mediated by direct activation of the phosphoinositide three (PI3) kinase signaling pathway through IGF-1 receptor/insulin receptor substrate 1 (IRS-1), but not by the ROL-RA pathway [32]. Several other reports have also discussed the existence of signaling pathways dependent on ROL, but distinct from that of RA. The hydroxylated forms of ROL were shown to regulate cell fate decisions by enhancing protein kinase C α (PKCα) and its downstream MAP kinase activity [33] or by supporting PDGF signaling [34]. Further studies are underway in our group to address whether these RA-independent mechanisms are also involved in ROL-mediated growth promotion in the proximal colonic stem cells. In addition, although the function of RA was not involved in ROL-dependent growth enhancement of proximal colon cells, RA induced a certain cellular response, an induction of the *Rarβ* gene, in cultured organoids. Interestingly, we showed that RA did not alter the expression level of *MUC2*, one of the marker genes that show cellular differentiation of colonic cells (Fig 4D). This argues against previous observations that RA promotes differentiation of colonic epithelial cells when laboratory adapted cell lines derived from human colon cancers were used as model systems [27, 28]. In this regard, it is also important to investigate what is the molecular function of RA in the physiological colonic epithelium and how RA influences cell fate determination of those epithelial cells.

Following the development of conditions for growing mouse small intestinal stem cells, the intestinal organoid system has been widely applied for various studies not only on the small intestine but also on other tissues including the colon [35, 36]. This methodological advance has led to new approaches to investigate the mechanism of proliferation of normal colonic epithelial cells [37–39] and also the pathways of their tumorigenesis [40–42]. To our knowledge, however, there has been no study that assessed *ex vivo* response of colonic epithelial stem cell populations of different parts along the length of the colon and described their different behaviors. In this context, our demonstration of the novel function of ROL on the growth of proximal colon organoids would not only evolve a better system to expand those cells with greater efficiency for many applications, but also stimulate research into the mechanism of region-specific regulation of colonic stem cell maintenance.

In summary, we showed that ROL has potent activity to promote the growth of proximal colon organoids by stimulating *Lgr5*+ stem cell populations. Further study to uncover this RA-independent mechanism would provide insights into the distinct features of proximal and distal colon stem cells.

## Supporting Information

S1 Table Information on primers and reaction conditions for PCR. Gene names, sequences of sense and antisense primers, and the cycle number to semi-quantitatively amplify each gene were presented.
(DOCX)
