# Supplementary material for: Retinol Promotes In Vitro Growth of Proximal Colon Organoids through a Retinoic Acid-Independent Mechanism
Source: PLoS One. 2016 Aug 26;11(8):e0162049. doi: 10.1371/journal.pone.0162049 (PMC5001647; doi:10.1371/journal.pone.0162049)
Supplement: S1 Table — Gene names, sequences of sense and antisense primers, and the cycle number to semi-quantitatively amplify each gene were presented. (DOCX) [file pone.0162049.s001.docx]

| Gene | Forward primer | Reverse Primer | Cycle number |
| --- | --- | --- | --- |
| *Lgr5* | AGATGCCAACCACATCAGCTAC | TCCACACTGTTGCCGTCGTCTT | 32 |
| *Adh1* | GCCGAAGCGATCTGCTAATG | AACGAAAAGTCCACCCCTCC | 26 |
| *Adh5* | ATCTTGGGACATGAAGGTGCTGGA | ACGTTGCCAATGCACTCAAAGGAG | 30 |
| *Adh7* | ATGGTTGATGCCCTCTCATC | GAACACCCAGGTCTCTGGAA | 32 |
| *Rdh1* | GAATTCTGCCAAGGGATTCA | CATCACCGGGAACTGAACCT | 30 |
| *Rdh10* | CACGCACACTTCTGGACCAC | GTACTGATTGATGCGCACGG | 30 |
| *Aldh1a1* | CACTGGCCGACTTGAAGATTCAAC | GAGTACTTCAAGGAGTCACTGAGG | 32 |
| *Aldh1a2* | TGAATGGCAGAACTCAGAGAGTGG | ATACTGTGGGTTGAAGGGAGCTAG | 32 |
| *Aldh1a3* | TTCCACGACATAGGCCCATCTAAG | ACACTACCTCCGTGTACTTACAGC | 32 |
| *Rarβ* | ATGTTCTGTCAGTGAGTCCC | TCTCTGTGCATTCCTGCTTTG | 35 |
| *Muc2* | TCCACCATGGGGCTGCCACT | GGCCCGAGAGTAGACCTTGG | 30 |
| *Gapdh* | CTGGCCAAGGTCATCCATGA | GCCATGAGGTCCACCACCCTG | 22 |

S1 Table. Information on primers and reaction conditions for PCR
